# Fluctuating Environments, Sexual Selection and the Evolution of Flexible Mate Choice in Birds

**DOI:** 10.1371/journal.pone.0032311

**Published:** 2012-02-16

**Authors:** Carlos A. Botero, Dustin R. Rubenstein

**Affiliations:** 1 National Evolutionary Synthesis Center, Durham, North Carolina, United States of America; 2 Department of Ecology, Evolution and Environmental Biology, Columbia University, New York, New York, United States of America; Dalhousie University, Canada

## Abstract

Environmentally-induced fluctuation in the form and strength of natural selection can drive the evolution of morphology, physiology, and behavior. Here we test the idea that fluctuating climatic conditions may also influence the process of sexual selection by inducing unexpected reversals in the relative quality or sexual attractiveness of potential breeding partners. Although this phenomenon, known as ‘ecological cross-over’, has been documented in a variety of species, it remains unclear the extent to which it has driven the evolution of major interspecific differences in reproductive behavior. We show that after controlling for potentially influential life history and demographic variables, there are significant positive associations between the variability and predictability of annual climatic cycles and the prevalence of infidelity and divorce within populations of a taxonomically diverse array of socially monogamous birds. Our results are consistent with the hypothesis that environmental factors have shaped the evolution of reproductive flexibility and suggest that in the absence of severe time constraints, secondary mate choice behaviors can help prevent, correct, or minimize the negative consequences of ecological cross-overs. Our findings also illustrate how a basic evolutionary process like sexual selection is susceptible to the increasing variability and unpredictability of climatic conditions that is resulting from climate change.

## Introduction

Local variation in precipitation and temperature (e.g., wet and dry periods, changing seasons, or El Niño events), can lead to temporal fluctuation in the form and strength of natural selection [1]–[5]. Such fluctuating selection (or oscillating selection, as in [1]) has been implicated in the maintenance of genetic variation under directional selection [1], and appears to be important for the evolution of morphology [1], cognition [6], [7], complex social behavior [8], [9], foraging flexibility [10], [11], and bet-hedging [12], [13]. Together, these studies suggest that fluctuating selection favors the evolution of more flexible traits by exposing individuals to a range of conditions rather than specific situations.

In addition to influencing the dynamics of natural selection, it has been suggested that temporal fluctuation in environmental conditions may also play a role in the process of sexual selection [14], [15]. In most sexual species, individuals attempt to maximize future direct or indirect benefits by choosing mates with superior phenotypic characteristics and/or more attractive courtship displays [16]. Because these secondary sexual characters are often plastic, sudden changes in environmental conditions can lead to unexpected reversals in the relative quality or sexual attractiveness of potential breeding options ([14], [17], [18] for review see [19]). Although this phenomenon, known as ecological cross-over [14], has been observed in a variety of species including mites [20], flies [21], birds [15], [22], fish [23], and frogs [24], it is currently unclear the extent to which it may explain general patterns of interspecific variation in reproductive behavior.

Populations exposed to frequent environmental variation may evolve flexible sexual preferences that allow individuals to select optimal partners in different ecological contexts. For example, female lark buntings, *Calamospiza melanocorys*, show highly variable sexual preferences among years and this variation appears to be driven by a stronger preference for the particular male trait that best predicts nesting success in any given year (e.g., beak size, wing patch size, or body color) [15]. However, when environmental change is sudden and unexpected, even species with highly flexible sexual preferences are likely to make mistakes. Furthermore, a higher prevalence of suboptimal partnerships can be expected in more variable and unpredictable habitats for at least three reasons: (1) when environmental conditions at the time of reproduction differ from those experienced during the production of sexual signals (or at the time in which females choose), courtship signals may be poor indicators of the direct benefits that a potential partner has to offer; (2) when conditions experienced by parents are likely to differ from those experienced by their offspring, current mate quality may be a poor indicator of future offspring viability (i.e., indirect benefits); and (3) when the conditions that offspring will experience are highly unpredictable, a greater genetic diversity among offspring could improve long-term reproductive success (e.g., number of grand-offspring), thereby favoring reproduction with multiple partners.

Given that suboptimal social partners can be highly detrimental to fitness, we hypothesized a link between environmental uncertainty and the prevalence of secondary mate choices among socially monogamous birds. To test this idea, we focus here on the rates of extra-pair mating (hereafter infidelity, [25]), and divorce [26]–[28], two types of secondary mate choices that have been sampled in a consistent way across a taxonomically diverse array of avian species (see Methods). Environmental uncertainty could influence the prevalence of these behaviors among socially monogamous birds in two alternative ways. One possibility is that environmental uncertainty increases the prevalence of infidelity and/or divorce relative to what would otherwise be expected from each species' life history and demography (for reviews on the effect of these variables on secondary mate choice see [25], [29], [30]). Although we acknowledge that divorce and infidelity may not be adaptive in every species (or even in both sexes, see [31]), this hypothesis assumes that, on average, these behaviors can prevent, correct, or minimize the negative consequences of suboptimal partnerships (see [25], [26], [30]). For example, extra-pair offspring tend to have higher survival rates [32], fledge in better condition [33], be more immunocompetent [34], and have higher lifetime reproductive success [35] than their within-pair half-sibs. Similarly, reproductive success and partner quality typically increase as a consequence of re-pairing after divorce [36]–[38]. An increase in the prevalence of infidelity and divorce is particularly likely when the frequency of environmental change is relatively low because under those conditions, individuals that have already experienced an ecological crossover may now acquire reasonably accurate information about the relative quality of available options in the new ecological setting. Alternatively, environmental uncertainty could lead to lower rates of infidelity and divorce by interfering with an individual's ability to determine if there are better reproductive options available. In many species, previous experience and familiarity with a breeding partner can lead to a measurable increase in reproductive success (see [26]). Because divorce and infidelity can compromise such benefits by straining or ending social partnerships, it may only be profitable for individuals to engage in these behaviors when it is highly likely that they will benefit from them. A decrease in the prevalence of infidelity and divorce is therefore likely when experience and familiarity with a partner are more important than mate quality for reproductive success, or when ecological cross-overs are frequent enough that conditions are likely to change again before a new partnership can be established. Thus, environmental uncertainty could potentially lead to either a decrease or an increase in infidelity or divorce, depending on the natural history of the species involved.

Here we explore the effects of environmental uncertainty on the evolution of reproductive behavior among socially monogamous birds. To estimate the extent to which populations are exposed to ecological cross-overs, we measure the variability and predictability of local temperature and precipitation patterns. After accounting for the potential effects of life history (i.e., adult mortality, ornamentation, dichromatism) and demography (i.e., continuity of partnerships, coloniality) [see 25,29,30], we demonstrate significant associations between environmental parameters and the incidence of avian infidelity and divorce. These findings illustrate how sexual selection may be susceptible to the globally increasing environmental uncertainty that is resulting from climate change (see [39], [40], [41]).

## Methods

### 1. Behavioral and Life History Variables

Infidelity is defined as cases in which at least some offspring in a brood are fathered by individuals other than the social partner [25]. We therefore measured this behavior as the proportion of nests in a population containing extra-pair young. Divorce is defined as cases in which individuals establish a breeding partnerships with a new mate even though their former partner is still alive and present in the population [26]. A comprehensive search of the literature published through 2010 yielded data on infidelity for 277 species and on divorce for 163 species. Sources were located via Web of Knowledge (http:/www.webofknowledge.com), Google Scholar (http://scholar.google.com/), and by direct inspection of the reference lists of major reviews on these topics. All secondary records were checked against primary sources for accuracy and to determine study locations. We geo-referenced all study sites using BioGeomancer WorkBench version 1.2.3 (http://www.biogeomancer.org) and confirmed every locality through direct inspection in Google Earth 5 (http://earth.google.com). We then computed climatic variables (see below for more detail) from the nearest weather station to each study site from the Global Historical Climatology Network (http://www.ncdc.noaa.gov) using a maximum cutoff distance of 100 km (great-circle distance between weather stations and study populations: Mode = 12.22 km; Mean = 31.15 km). In addition, we used molecular data from GenBank to build independent phylogenetic hypotheses for our comparative analyses (see below for more detail). Because climatic or molecular data were not available for every species, our final sample includes 122 species for infidelity and 86 species for divorce (Table S1). We analyze these two datasets separately because infidelity and divorce have been previously linked to different causal factors(see [25], [26]), and because in our sample, there is no evidence of a correlation between these behaviors (Pearson's product-moment correlation of phylogenetically independent contrast: t = 0.6122, df = 22, P = 0.547) (but see [42]).

In exploring the effects of climatic uncertainty on reproductive flexibility, we also consider the roles of other potential covariates such as life history and demography. Several variables are thought to be important for the evolution of infidelity and divorce and have received different levels of support in analyses of single species or closely-related groups. To avoid overparameterization of our models, we consider only those variables that explain broad-scale, interspecific differences in previously published studies. Earlier comparative analyses have failed to find any strongly supported predictors in the case of avian infidelity, but breeding density, parental care, and adult mortality appear to have at least some explanatory power (see [25], [30]). Specifically, higher frequencies of extra-pair mating are expected under higher breeding densities because physical proximity may increase the availability of alternative reproductive options and the opportunity for extra-pair copulation [43]. Similarly, a higher incidence of extra-pair mating is expected when females are able to rear offspring on their own because they have little to loose if their cuckolded partners retaliate with a reduction in parental care [44]. In addition, extra-pair mating is expected to be higher in species with shorter lifespans because when future reproductive opportunities are unlikely, abandoning a clutch or withholding parental care in response to infidelity is expected to be highly maladaptive [45]. Our models therefore include coloniality (as a proxy for breeding density) and adult mortality (measured as the per-year probability of dying for both sexes combined) as potential predictors of avian infidelity, and control for the mode of parental care by including only species that are socially monogamous, show bi-parental care, and do not breed in social or cooperatively breeding groups. Note that the metric of adult mortality in this model (as well as in the model of divorce; see below) captures the range of fast/slow life history strategies that is known to influence reproductive decisions [46].

In the case of avian divorce, our analysis is informed by a recent comparative analysis that found support for 5 of 19 predictors gathered from the literature [29]. The well-supported predictors of divorce are continuity of partnerships (i.e., whether partners remain together year-round or separate during the non-breeding season), sexual dichromatism, visual ornamentation (scale from 0 = not ornamented, to 7 = very ornamented; data from [28] when available, missing species measured using identical protocols by JF, see acknowledgements, and CAB), coloniality, and adult mortality. Divorce rates are expected to be higher in species with stronger sexual selection (i.e., more visually ornamented or more sexually dichromatic) because of stronger competition for high quality mates [28]. Similarly, divorce is expected to be more common in species with high mortality rates because delaying breeding in order to wait for partners that may never come back can be prohibitively costly (also a likely explanation for the effect of separating during the non-breeding season); or because in short-lived species, it is more likely that higher quality mates will become available through the death of their own partners (see [29]). In addition to these five predictors, we include in this model two-way interactions that explore the potentially different effects of environmental variability on species that establish continuous versus temporary partnerships. Such interactions are biologically meaningful because individuals that stay together year-round are constantly aware of each other's status and do not incur searching or waiting costs at the onset of new breeding opportunities [26].

### 2. Climatic Variables

From each local weather station included in this study we obtained monthly averages of precipitation and temperature retaining only years with complete records from 1800 to 2009. Because this timeframe captures only fairly recent events in evolutionary history, the climate variables described below reflect the ecological conditions (or ‘climatic tolerances’) to which the species in this study are currently adapted (just as our metrics of infidelity and divorce reflect current levels of secondary mate choice in this group). Raw climate data were used to compute the following descriptive measures of the local variation in precipitation and temperature at each site: (1) within-year variance (i.e., variance in the monthly means for each year then averaged across years); (2) predictability of annual cycles (see below); (3) mean for the breeding season (see below); and (4) among-year variance in the mean of the breeding season. The predictability of annual climate cycles was quantified via Colwell's *P* [47] using standard bin sizes of 50 mm for precipitation and 0.5 degrees Celsius for temperature. Colwell's P is an information-theory-based index that can be interpreted as a measure of the extent to which the onset, duration, and intensity of local climate cycles differ among years. Sites with highly repeatable annual cycles yield P≈1 whereas those in which climate cycles show considerable variation among years yield P≈0.

Because of a lack of data on the phenology of most populations included in this study, we estimated indirectly the timing and duration of the breeding season at each locality through le Houerou's [48] mean growing season formula. This formula identifies the months of the year in which environmental conditions allow significant plant productivity, considered here to be a proxy for suitable breeding conditions. Although the timing and duration of the breeding season are also likely to depend on species-specific factors, le Houerou's formula is appropriate for an analysis at this scale because it provides a realistic, albeit coarse approximation of the months of the year in which reproduction is more likely to occur in different parts of the World. Specifically, the growing season months at a given site are defined as those in which the average daily temperature is greater than or equal to a critical threshold (0°C for arctic and boreal regions [49], 6°C for temperate and subtropical regions [50], and 12°C for the tropics [51]) and the total precipitation in millimeters is at least twice the average temperature in degrees centigrade. The mean growing season formula has been used and validated in comparative studies at a global scale in the field of evolutionary anthropology [e.g., 50].

### 3. Statistics

We used phylogenetic generalized least squares (PGLS) regression models with Pagel's λ [52] to account for the potential non-independence of data from species that vary in levels of relatedness [53]. Higher λ values (estimated from the data via restricted maximum likelihood [54]) indicate stronger similarities between closely related taxa [52]. Pagel's λ models are mathematically equivalent to ordinary least squares regression analysis (i.e., no phylogenetic correction) when λ = 0, and to PGLS regression analysis with a Brownian model of evolution when λ = 1. Logarithmic or Arcsine transformations of raw variables were applied as needed to meet the assumptions of additive linear relationships and multivariate normality in PGLS. All continuous predictors were then standardized prior to analysis to facilitate comparison of their relative effects. Models were run using the MATLAB routine REGRESSIONv2.m described in [54]. We began each analysis with a fully parameterized model and proceeded to drop each non-significant term one at a time, starting with the interactions when available. Finally, we computed variance inflation factors (VIF) for each significant predictor in the reduced final model to confirm that there were no major multicollinearity issues (VIF>5 are considered evidence of multicollinearity, see [55]). We report the R^2^ (computed as in equation 2.3.16, p. 32, from [56]), the estimated Pagel's lambda, and the β coefficients and test statistics for each significant term in our final models.

The phylogenetic hypotheses used in our comparative analyses (Figs. S1 and S2) are based on species-level molecular phylogenies generated from both nuclear and mitochondrial DNA sequences (see Table S1). When molecular data were not available for a species that was the only member of its genus in our sample (Infidelity dataset: *N* = 3/122; divorce dataset: *N* = 3/86), we used instead sequences from available congeners. DNA sequences were downloaded from GenBank (http://www.ncbi.nlm.nih.gov/Genbank), aligned using MUSCLE [57], and concatenated using Phyutility [58]. Phylogenies were estimated in RAxML 7.0.4 [59] using maximum likelihood, a General Time Reversible model of nucleotide substitution with a Γ model of rate heterogeneity, and topological constraints from Hackett et al. [60]. The resulting trees were ultrametrisized using Sanderson's [61] algorithm for molecular dating with penalized likelihood.

## Results

**Figure 1:**
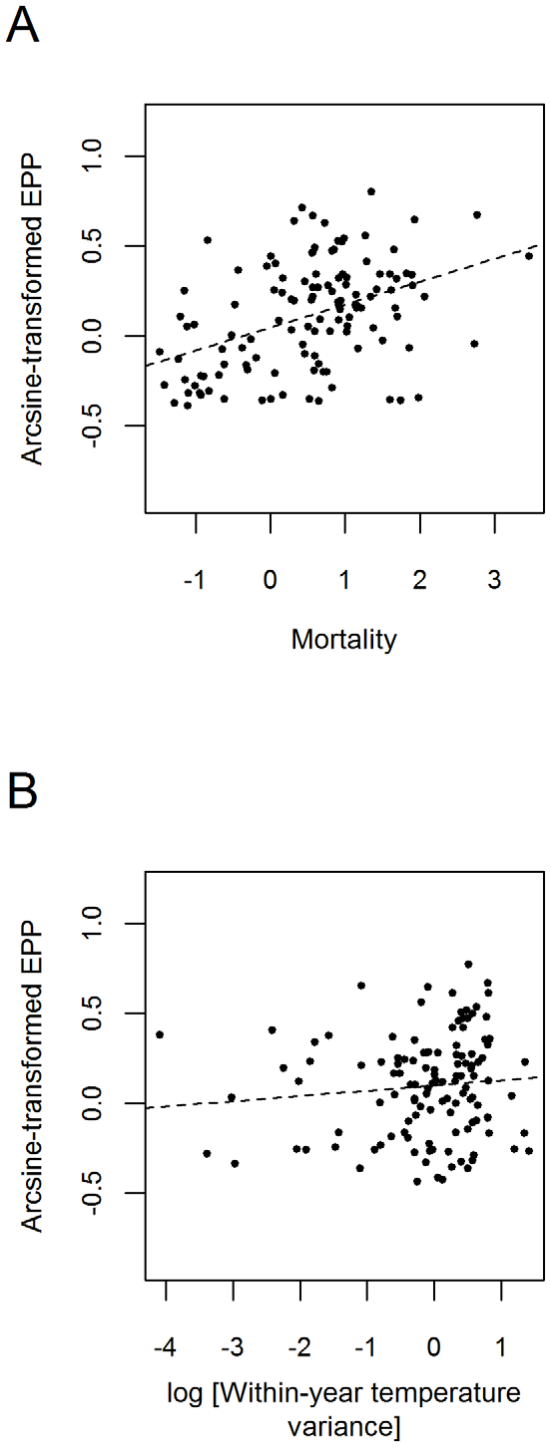
Partial regression plots of the statistically significant predictors of avian infidelity. Axes refer to multivariate residuals after correcting for phylogeny and the effects of other significant parameters in the model. Each data point represents one species and dotted lines depict linear trends in the relationships between variables.

Our fully parameterized model of avian infidelity includes coloniality, adult mortality, and climate variables (see Methods) as potential predictors. After removing all non-significant terms from this model (final PGLS regression model: *R* ^2^ = 0.08, λ = 0.55; VIF = 1.22, see [55]), we find that extra-pair broods are more common in species that experience higher adult mortality (β = 0.050, t = 2.056, df = 119, P = 0.042; Fig. 1A), and breed in environments with greater within-year variance in temperature (β = 0.042, t = 2.068, df = 119, P = 0.041; Fig. 1B).

**Figure 2:**
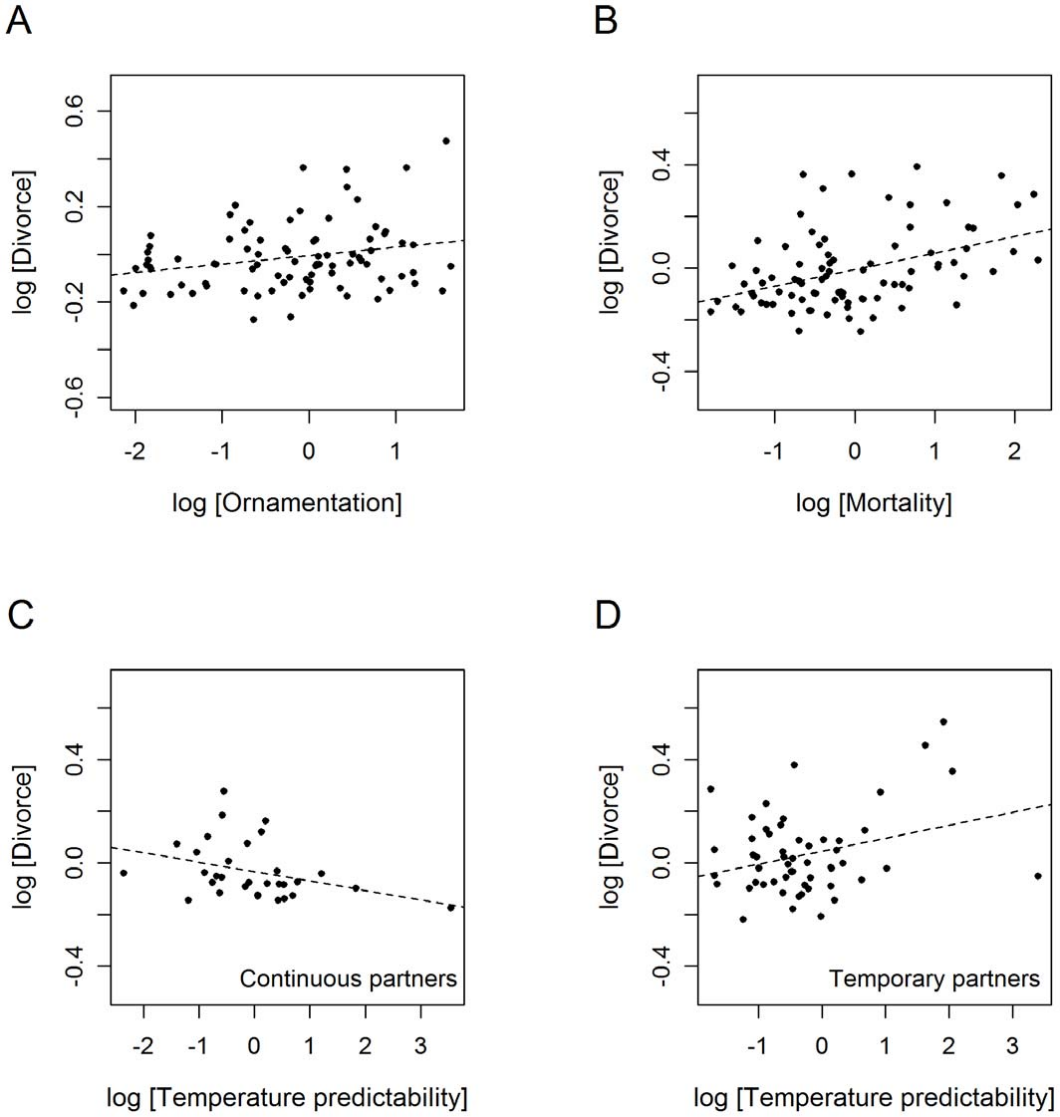
Partial regression plots of the statistically significant predictors of avian divorce. Axes refer to multivariate residuals after correcting for phylogeny and the effects of other significant parameters in the model. Each data point represents one species and dotted lines depict linear trends in the relationships between variables.

Our fully parameterized model of avian divorce includes coloniality, mortality, continuity of partnerships, sexual dichromatism, visual ornamentation, climate variables, and the two-way interactions between partnership continuity and the climate variables. Once non-significant terms are removed from this model (final PGLS regression model: *R* ^2^ = 0.34, λ = 0.65; all VIF<1.54, see [55]), we find that the frequency of divorce is higher in species with more pronounced visual ornamentation (β = 0.034, t = 2.348, df = 80, P = 0.021; Fig. 2A) and higher adult mortality (β = 0.089, t = 5.182, df = 80, P<0.001; Fig. 2B). In addition, we find that the frequency of divorce is also affected by the interaction between continuity of partnerships and predictability of annual temperature cycles (continuity: β = 0.001, t = 0.021, df = 80, P = 0.984; predictability of annual temperature cycles: β = −0.058, t = −2.279, df = 80, P = 0.025; continuity×temperature predictability: β = 0.112, t = 3.604, df = 80, P = 0.001); species that maintain year-long partnerships exhibit higher rates of divorce in more unpredictable environments (Fig. 2C), whereas those that engage only in temporary partnerships exhibit the opposite pattern (Fig. 2D).

## Discussion

Through a comparative analysis of the rates of infidelity and divorce in socially monogamous birds, we found that the process of sexual selection is likely to be influenced not only by intrinsic life history and demographic variables, but also by extrinsic factors such as the variability and predictability of local climates. As noted in earlier studies [25], [29], we find positive associations between adult mortality and secondary mate choices in socially monogamous birds both within (Fig. 1A) and among breeding seasons (Fig. 2B). These results are in agreement with previous findings that suggest that shorter lifespans lead to a lower tolerance of suboptimal partnerships because the expectation of future breeding opportunities is relatively low (see [26] for review). Similarly, we find that divorce is more common in more ornamented species (Fig. 2A, also noted in [29]), which supports the idea that strong sexual selection can promote the continued search for better reproductive options even after the establishment of breeding pair-bonds [62]. After accounting for these intrinsic factors, we find that the prevalence of infidelity is also positively associated with environmental variability. Although our model of infidelity explains a relatively small amount of the variation compared to that of divorce, the results are consistent with the genetic diversity hypothesis, which posits that more variable environments should favor behaviors that increase the genetic diversity among offspring whenever different environmental conditions favor different genotypes [63]–[66]. Additionally, our data show that the prevalence of divorce is also influenced by climatic factors, but in this case we find that it is associated with the predictability rather than with the variability of the environment. In other words, while the range of possible environmental conditions appears influence the expression of infidelity, it is the way in which different environmental conditions occur (e.g., the variation among years in the onset, intensity, or duration of environmental cycles) what matters most for divorce. The negative slope in Fig. 2C suggests that the potential for divorce increases when ecological cross-overs are more likely, which is consistent with the hypothesis that species that engage in continuous partnerships use divorce to help prevent, correct, or minimize the negative fitness consequences of suboptimal partnerships [26], [32]–[38]. In contrast, the positive slope in Fig. 2D indicates that temporary partners may experience greater difficulty in detecting or achieving better reproductive options in less predictable environments. In our sample, temporary partners are exposed to much shorter breeding seasons than year-round partners (PGLS: R^2^ = 0.07, λ = 0.17, t = −2.418, df = 84, P = 0.018), meaning that they are likely to experience important fitness losses even from minor delays in egg laying. Because locating and attracting a more suitable partner after divorce can take time (see [26]), this behavior is especially likely to experience strong negative selection in species with very short and unpredictable breeding opportunities. Thus, the positive association between the predictability of annual climatic cycles and divorce observed in this group suggests a partial release from time constraints in environments where the onsets of breeding opportunities are easier to predict.

Although our data suggest that the rates of infidelity and divorce are affected by temperature cycles, we find no evidence that they are affected by local variation in precipitation. This result suggests that the patterns we uncovered here may not be driven by changes in resource abundance, which are typically determined by rainfall (e.g., [67], [68]). For example, our sample in the divorce dataset includes several seabirds whose breeding success can be directly affected by changes in temperature but not necessarily by the existence of resource pulses on land (because these species forage at sea). Alternatively, the lack of an effect of precipitation could simply reflect the unfortunate geographic bias in the availability of samples for infidelity and divorce. In particular, most studies on these behaviors have been conducted in temperate, boreal, and polar regions (see Figs. S3 and S4), where the patterns of precipitation are fairly predictable (compared to the arid and semi-arid tropics) and the main source of environmental uncertainty is the variation in temperature (see Fig. 2 in [8]).

It could also be argued that our results are not a product of interspecific differences in exposure to ecological cross-overs, but rather due to intrinsic differences in cognitive ability. Given the positive association between environmental uncertainty and cognition [6], [7], [69], it is possible that species exposed to more variable and unpredictable conditions engage more often in divorce and infidelity because they are better able to assess the relative value of available reproductive options. As a preliminary test of this hypothesis, we used the residuals of a log-log regression of brain mass on body mass (i.e., relative brain size) as a proxy for relative cognitive ability (see Text S1 and [70], [71]) and repeated our analysis on the subset of species for which brain size data are available in the literature (Infidelity: N = 101; Divorce: N = 66, see Table S1). However, we found no evidence of an effect of relative brain size on infidelity (PGLS: t = 0.847, df = 97, P = 0.399), or divorce (PGLS: t = −0.185, df = 59, P = 0.854) and therefore, we conclude that differences in cognitive ability are unlikely to explain all aspects of the association between climatic tolerances and these behaviors.

In conclusion, our results are consistent with the idea that environmental factors shape the evolution of reproductive behavior and influence the process of sexual selection [14], [72], [73]. Our data suggest that although these extrinsic factors have likely played a stronger role in the evolution of divorce than infidelity, they nevertheless are able to account for a non-trivial amount of interspecific variation in both of these behaviors. We conclude that in the absence of severe time constraints, the selection of mates is aided by the facultative expression of secondary mate choices that correct or minimize the negative consequences of unexpected ecological cross-overs. Future research should evaluate whether current levels of reproductive flexibility determine a species' ability to respond to the global increase in environmental uncertainty that is resulting from recent climate change [39]–[41].

## Supporting Information

Figure S1 **Maximum likelihood phylogeny for 122 species included in our analysis of avian infidelity.**
(PDF)

Figure S2 **Maximum likelihood phylogeny for 86 species included in our analysis of avian divorce.**
(PDF)

Figure S3 **Global distribution of study sites in the infidelity dataset.**
(PDF)

Figure S4 **Global distribution of study sites in the divorce dataset.**
(PDF)

Text S1 Log-log regression model of the association between brain volume and body size.
(PDF)

Table S1 Raw data and data sources.
(XLS)
