# Supplementary material for: Fluctuating Environments, Sexual Selection and the Evolution of Flexible Mate Choice in Birds
Source: PLoS One. 2012 Feb 16;7(2):e32311. doi: 10.1371/journal.pone.0032311 (PMC3281128; doi:10.1371/journal.pone.0032311)
Supplement: Figure S1 — Maximum likelihood phylogeny for 122 species included in our analysis of avian infidelity. (PDF) [file pone.0032311.s001.pdf]

Supporting Information

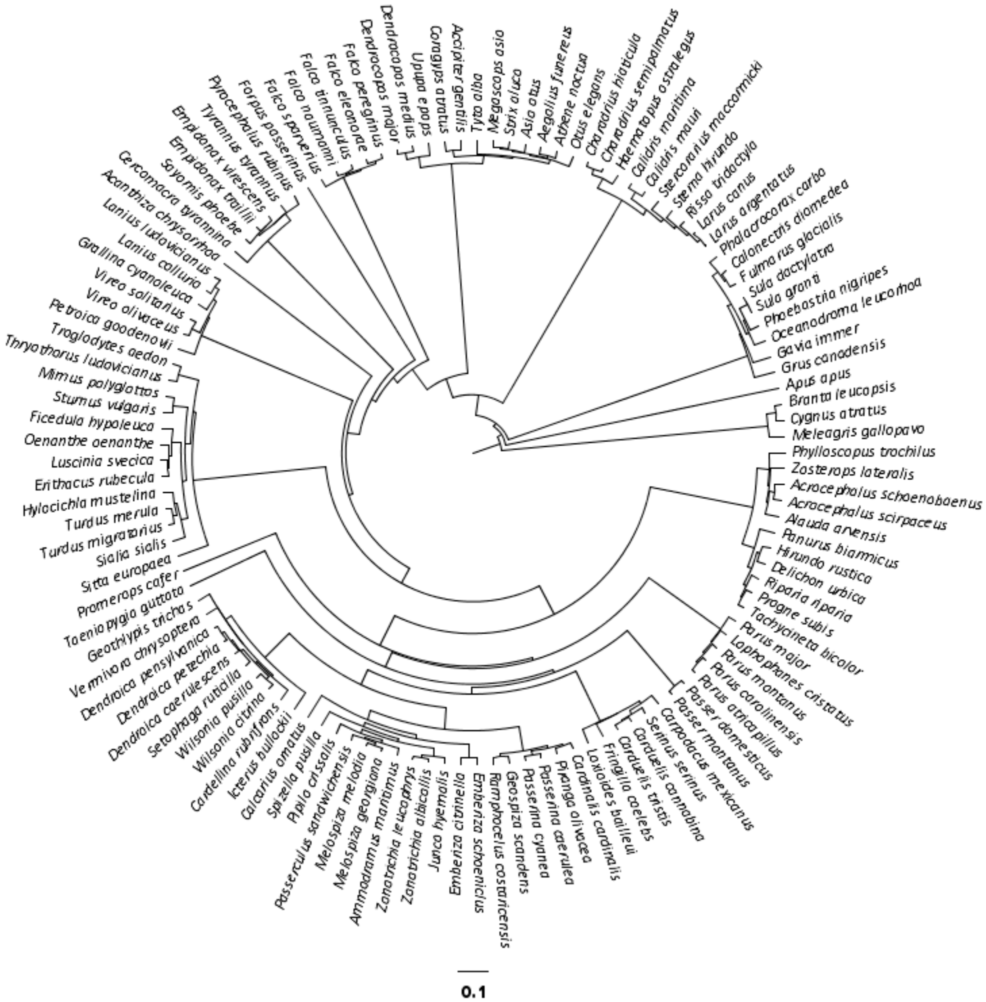

Figure S1. Maximum likelihood phylogeny for 122 species included in our analysis of avian infidelity
