# Supplementary figures and images for: Fluctuating Environments, Sexual Selection and the Evolution of Flexible Mate Choice in Birds
Source: PLoS One. 2012 Feb 16;7(2):e32311. doi: 10.1371/journal.pone.0032311 (PMC3281128; doi:10.1371/journal.pone.0032311)

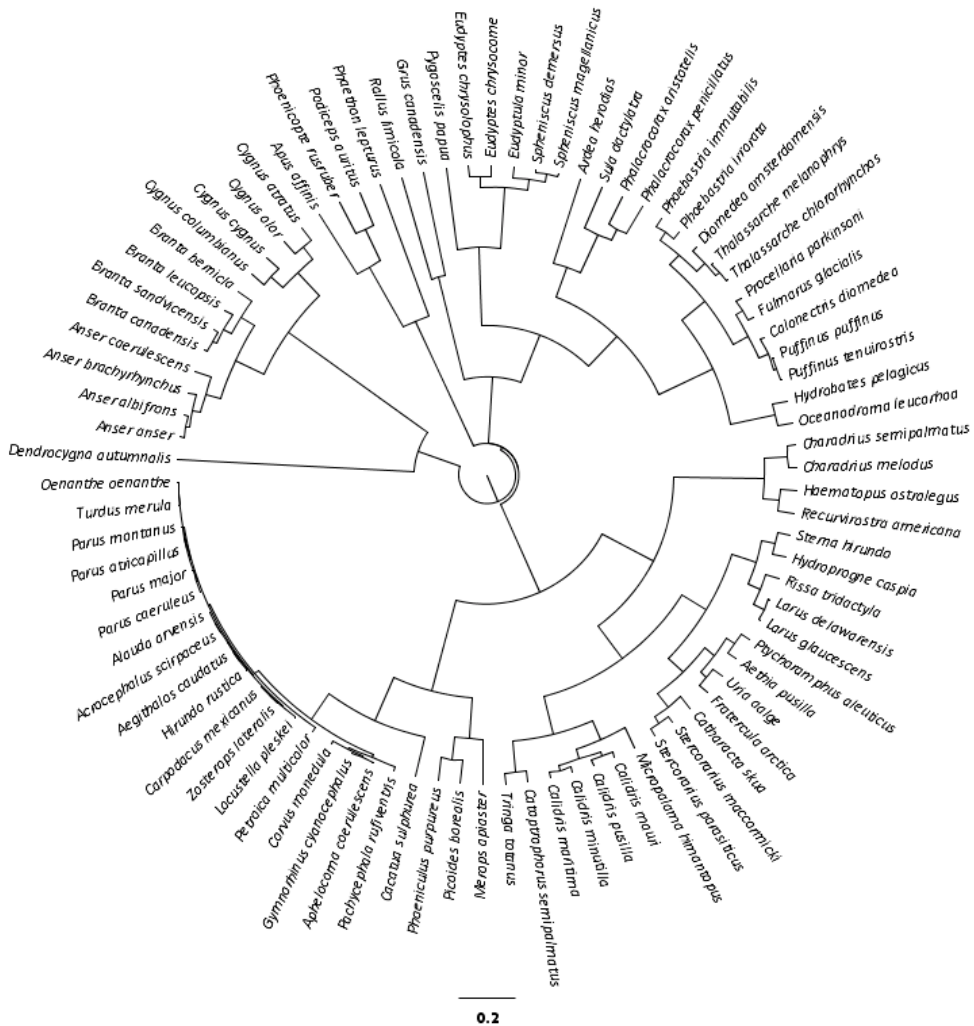

**Figure S2. Maximum likelihood phylogeny for 86 species included in our analysis of avian divorce**

Supplement: Figure S2 — Maximum likelihood phylogeny for 86 species included in our analysis of avian divorce. (PDF) [file pone.0032311.s002.pdf]

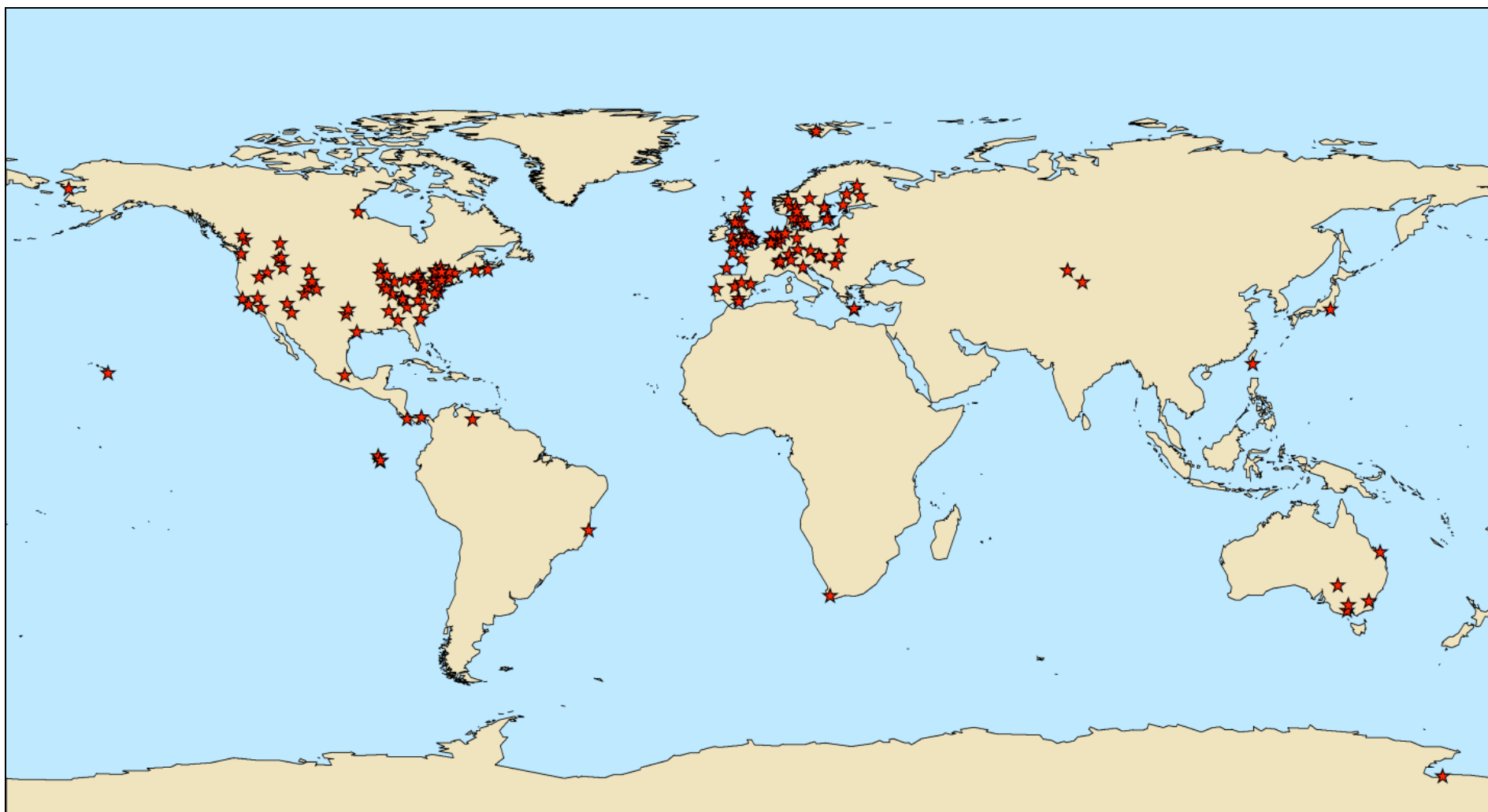

**Figure S3** Global distribution of study sites in the infidelity dataset.

Supplement: Figure S3 — Global distribution of study sites in the infidelity dataset. (PDF) [file pone.0032311.s003.pdf]

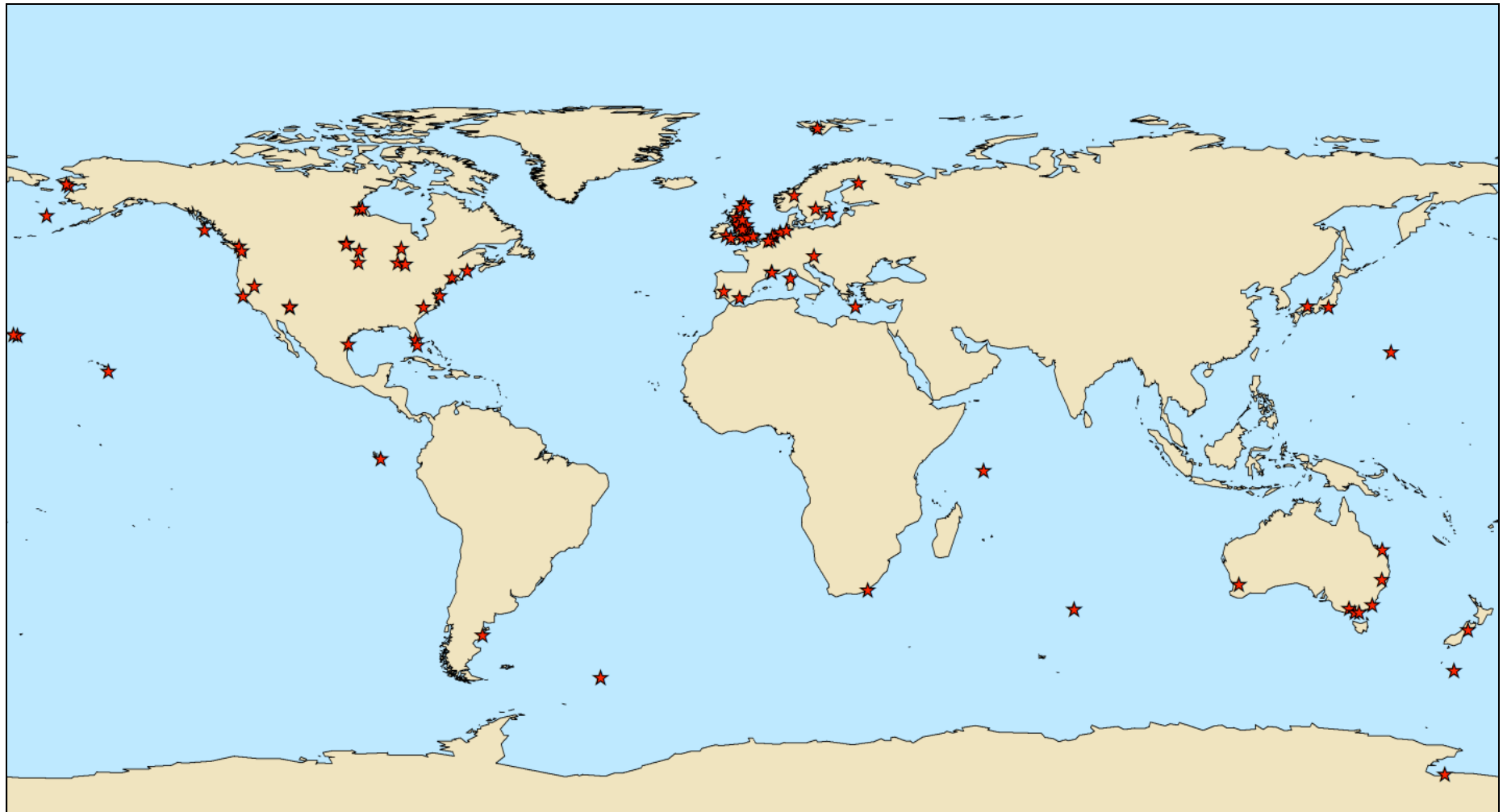

**Figure S4** Global distribution of study sites in the divorce dataset.

Supplement: Figure S4 — Global distribution of study sites in the divorce dataset. (PDF) [file pone.0032311.s004.pdf]
