# Supplementary material for: Fluctuating Environments, Sexual Selection and the Evolution of Flexible Mate Choice in Birds
Source: PLoS One. 2012 Feb 16;7(2):e32311. doi: 10.1371/journal.pone.0032311 (PMC3281128; doi:10.1371/journal.pone.0032311)
Supplement: Text S1 — Log-log regression model of the association between brain volume and body size. (PDF) [file pone.0032311.s005.pdf]

Text S1. Log-log regression model of the association between brain volume and body size

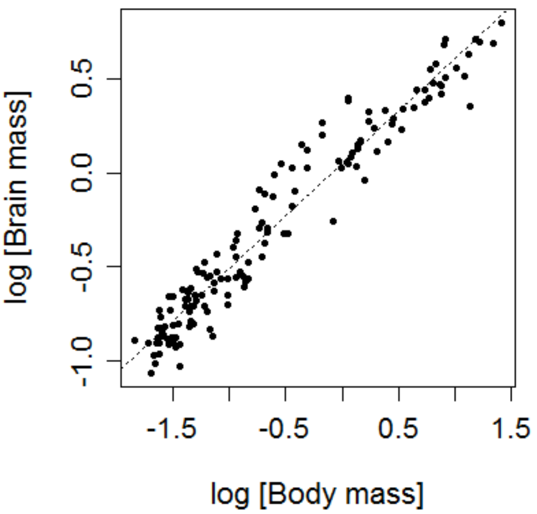

Because brain size scales allometrically, we used the residuals of a log-log regression of brain volume on body size as a proxy for cognitive ability (see [1, 2]). Data on whole brain mass were available for 89 species and data on whole brain volume were available for an additional 57 species in our combined datasets. We used the density of fresh brain tissue (1.036 g · ml^{-1}) to convert volumes to mass [3], yielding a total sample size of 145 species, 101 in the infidelity dataset and 66 in the divorce dataset (see Table S1 in Electronic Supplementary Material for data and data sources). As expected, body size accounts for most of the interspecific variation in brain volume (PGLS: $R^2 = 0.94$, $\lambda = 0.00$, $\beta = 0.558$, $t = 47.501$, $df = 143$, $P < 0.001$). The residuals from this regression were labeled 'relative brain size' and this new variable was used as a proxy for cognitive ability in the models presented in the main text.

References

1. Bennett P.M., Harvey P.H. 1985 Relative brain size and ecology in birds. *J Zool* **207**(OCT), 151-169.

2. Deaner R.O., Nunn C.L. 1999 How quickly do brains catch up with bodies? A comparative method for detecting evolutionary lag. *Proceedings of the Royal Society of London Series B-Biological Sciences* **266**(1420), 687-694.

3. Sol D., Lefebvre L., Rodriguez-Teijeiro J.D. 2005 Brain size, innovative propensity and migratory behaviour in temperate Palaearctic birds. *Proceedings of the Royal Society B-Biological Sciences* **272**(1571), 1433-1441.
